# Assessment of local treatment modalities for FIGO stage IB-IIB cervical cancer: A propensity-score matched analysis based on SEER database

**DOI:** 10.1038/s41598-017-03580-5

**Published:** 2017-06-09

**Authors:** Xing Song, Yang Han, Yingjie Shao, Wendong Gu, Honglei Pei, Jingting Jiang

**Affiliations:** 1 Department of Radiation Oncology, The Third Affiliated Hospital of Soochow University, 185 Juqian Street, Changzhou, 213003 People’s Republic of China; 2 Department of Tumor Biological Treatment, The Third Affiliated Hospital of Soochow University, 185 Juqian Street, Changzhou, 213003 People’s Republic of China

## Abstract

The aim of this study was to investigate the impact of local treatment modalities on the survival of patients with International Federation of Gynecology and Obstetrics (FIGO) stage IB-IIB cervical cancer, including cancer-directed surgery (CDS) alone and CDS combined with radiotherapy (RT). A total of 8,357 patients with cervical cancer between 1988 and 2013 were included in the final study cohort, including 4,298 (51.4%) patients who underwent CDS alone and 4,059 (48.6%) patients who received combination therapy. Univariate and multivariate analyses showed that local treatment modalities were prognostic factors for cause-specific survival (CSS). Patients who received combination therapy had worse CSS (HR = 1.38; 95% CI = 1.20–1.59; *P* < 0.001). Subgroup analyses showed the prognostic effect of local treatment modalities was significantly influenced by FIGO stage. In the propensity-score matched (PSM) dataset, CDS was associated with better CSS (*P* < 0.001) for patients with IB-IIA cervical cancer; nevertheless, no differences were observed in CSS (*P* = 0.639) for patients with IIB cervical cancer. In conclusion, radical surgery was the preferred treatment for patients with IB-IIA cervical cancer, and there was no difference between radical surgery alone and combination therapy for patients with IIB cervical cancer.

## Introduction

Despite the significant advances in the screening and treatment of cervical dysplasia, cervical cancer is the sixth most prevalent female malignancy, and the incidence and mortality of cervical cancer is increasing yearly in China^1, 2^. According to the National Comprehensive Cancer Network (NCCN) guidelines, the current standard treatments for International Federation of Gynecology and Obstetrics (FIGO) stage IB-IIB cervical cancer are recommended according to disease stages^3^. For most patients presenting with IB-IIA disease, radical hysterectomy and pelvis with or without para-aortic lymphadenectomy is the current standard treatment, and no differences were observed between radiotherapy and surgery in terms of 5-year survival rates. Additionally, for patients with IIB cervical cancer, combination therapy is the standard treatment method^4^.

In practice, there is still controversy on choice of treatment method for early stage cervical cancer. Several studies suggested that radical surgery should be enough for local disease control even without adjuvant or neoadjuvant radiotherapy (RT)^5–7^. However, it has also been suggested the survival for combination therapy is superior to primary surgery alone^8–10^. Unfortunately, it is difficult to draw conclusions on local treatments in early stage cervical cancer from previous studies comprised of limited case reports^11^. In this regard, we used the data from the Surveillance Epidemiology and End Results (SEER) database to investigate the difference between combination therapy and primary surgery alone. Furthermore, we discovered significant differences in the baseline characteristic of previous studies. Thus, in this study, we performed propensity-score matched (PSM) analyses to adjust for biases from baseline characteristics of the two treatment groups.

## Results

### Baseline characteristics in the entire population

In the unmatched database, a total of 8,357 patients met our inclusion criteria and were included in our final analysis. The median follow-up time was 72 months (range: 0–311 months). Table 1 summarizes the characteristics of the study population. The median age of cervical cancer diagnosis was 43 years (range: 15–97 years) and 31.8% of patients were aged 50 years or more. A total of 4,298 (51.4%) patients underwent CDS and 4,059 (48.6%) patients received CDS + RT treatment. Significant differences between two treatment groups were recorded regarding patient characteristics. As shown in Table 1, it was investigated that significant differences were found among the age, race, marital status, histological subtypes, primary site, grade, FIGO stage, nodal status size and surgical methods. All of them had statistical difference (*P* < 0.05) except race (*P* = 0.072) and marital status (*P* = 0.138).

**Table 1:** Characteristics of Patients.

Variable	n	CDS + RT (%)	CDS (%)	P value
		(n = 4059)	(n = 4298)	
Age				<0.001
<50	5697	2558 (63.0)	3139 (73.0)	
≥50	2660	1501 (37.0)	1159 (27.0)	
Race				0.072
White	6587	3166 (78.0)	3421 (79.6)	
Black	775	406 (10.0)	369 (8.6)	
Other	995	487 (12.0)	508 (11.8)	
Marital status				0.138
Single	2121	1008 (24.8)	1113 (25.9)	
Married	6236	3051 (75.2)	3185 (74.1)	
Histologic type				<0.001
Squamous cell carcinoma	5137	2673 (65.9)	2464 (57.3)	
Adenocarcinoma	1363	508 (12.5)	855 (19.9)	
Adenosquamous carcinoma	565	301 (7.4)	264 (6.1)	
Others	1292	577 (14.2)	715 (16.6)	
Primary site				0.001
Cervix uteri	5840	2917 (71.9)	2923 (68.0)	
Endocervix	1986	899 (22.1)	1087 (25.3)	
Exocervix	236	101 (2.5)	135 (3.1)	
Overlapping lesion of cervix uteri	295	142 (3.5)	153 (3.6)	
Grade				<0.001
I	1061	330 (8.1)	731 (17.0)	
II	3539	1612 (39.7)	1927 (44.8)	
III	3513	1969 (48.5)	1544 (35.9)	
IV	244	148 (3.6)	96 (2.2)	
FIGO				<0.001
IB	6577	2561 (63.1)	4016 (93.4)	
IIA	391	299 (7.4)	92 (2.1)	
IIB	1389	1199 (29.5)	190 (4.4)	
Nodal status				<0.001
Node negative	6586	2533 (62.4)	4053 (94.3)	
Node positive	1771	1526 (37.6)	245 (5.7)	
Size				<0.001
≤2 cm	3350	929 (22.9)	2421 (56.3)	
2–4 cm	3044	1637 (40.3)	1407 (32.7)	
>4 cm	1963	1493 (36.8)	470 (11.0)	
Surgical method				<0.001
Local tumor destruction	800	582 (14.3)	218 (5.1)	
Hysterectomy	7472	3403 (83.8)	4069 (94.7)	
Pelvic exenteration	85	74 (1.8)	11 (0.3)	

CDS: cancer-directed surgery; RT: radiotherapy; FIGO: International Federation of Gynecology and Obstetrics.

### Analysis of prognostic factors

In the dataset, univariate analysis showed that age, race, histological subtypes, primary site, grade, FIGO stage, nodal status, tumor size, surgical methods and local treatment modalities were prognostic factors that affected CSS (*P* < 0.05; Table 2). Marital status was not associated with CSS (*P* > 0.05; Table 2). Multivariate analysis showed that race, histological subtypes, grade, FIGO stage, nodal status, tumor size, surgical methods and local treatment modalities were prognostic factors that affected CSS (*P* < 0.05; Table 3). Patients who received CDS + RT treatment had worse CSS (HR = 1.38; 95% CI = 1.20–1.59; *P* < 0.001).

**Table 2:** Univariate and multivariate analysis of cause-specific survival.

Variable	Univariate	Multivariate		
	P	HR	95%	P
Age ≥ 50	0.001	1.02	0.91–1.15	0.708
Race	<0.001			<0.001
White		1		
Black		1.45	1.23–1.71	<0.001
Other		1.13	0.96–1.33	0.138
Married patients	0.906	—	—	—
Histologic type	<0.001			<0.001
Squamous cell carcinoma		1		
Adenocarcinoma		1.47	1.23–1.76	<0.001
Adenosquamous carcinoma		1.29	1.06–1.58	0.011
Others		1.74	1.47–2.05	<0.001
Primary site	0.002			0.120
Cervix uteri		1		
Endocervix		0.88	0.76–1.03	0.104
Exocervix		0.77	0.54–1.09	0.136
Overlapping lesion of cervix uteri		0.81	0.60–1.10	0.185
Grade	<0.001			<0.001
I		1		
II		1.89	1.44–2.48	<0.001
III		2.60	1.98–3.41	<0.001
IV		3.11	2.19–4.42	<0.001
FIGO	<0.001			<0.001
IB		1		
IIA		1.83	1.51–2.23	<0.001
IIB		1.75	1.52–2.00	<0.001
Node positive	<0.001	1.90	1.68–2.14	<0.001
Size	<0.001			<0.001
≤2 cm		1		
2–4 cm		1.93	1.64–2.27	<0.001
≥4 cm		2.53	2.13–3.01	<0.001
Surgical method	<0.001			<0.001
Local tumor destruction		1		
Hysterectomy		0.80	0.68–0.96	0.014
Pelvic exenteration		1.45	1.03–2.03	0.033
CDS + RT	<0.001	1.38	1.20–1.59	<0.001

HR = hazard ratio; CI = confidence interval; FIGO: International Federation of Gynecology and Obstetrics; CDS: cancer-directed surgery; RT: radiotherapy.

**Table 3:** Baseline characteristics of PSM cohorts for FIGO stage IB-IIA.

Variable	Unmatched (complete) dataset			Matched (1:1) dataset		
	CDS (%)	CDS + RT (%)	P	CDS (%)	CDS + RT (%)	P
	(n = 4108)	(n = 2860)		(n = 1499)	(n = 1499)	
Age			<0.001			0.938
<50	3052 (74.3)	1897 (66.3)		994 (66.3)	996 (66.4)	
≥50	1056 (25.7)	963 (33.7)		505 (33.7)	503 (33.6)	
Race			0.117			0.912
White	3275 (79.7)	2253 (78.8)		1232 (82.2)	1223 (81.6)	
Black	344 (8.4)	280 (9.8)		131 (8.7)	135 (9.0)	
Other	489 (11.9)	327 (11.4)		136 (9.1)	141 (9.4)	
Marital status			0.213			0.791
Single	1060 (25.8)	713 (24.9)		329 (21.9)	323 (21.5)	
Married	3048 (74.2)	2147 (75.1)		1170 (78.1)	1176 (78.5)	
Histologic type			<0.001			0.999
Squamous cell carcinoma	2329 (56.7)	1861 (65.1)		1103 (66.9)	1000 (66.7)	
Adenocarcinoma	843 (20.5)	369 (12.9)		204 (13.6)	206 (13.7)	
Adenosquamous carcinoma	249 (6.1)	213 (7.4)		83 (5.5)	84 (5.6)	
Others	687 (16.7)	417 (14.6)		209 (13.9)	209 (13.9)	
Primary site			0.033			0.986
Cervix uteri	2771 (67.5)	2108 (70.6)		1084 (72.3)	1080 (72.0)	
Endocervix	1053 (25.6)	661 (23.1)		341 (22.7)	348 (23.2)	
Exocervix	134 (3.2)	76 (2.7)		34 (2.3)	33 (2.2)	
Overlapping lesion of cervix uteri	150 (3.7)	105 (3.7)		40 (2.7)	38 (2.5)	
Grade			<0.001			0.988
I	725 (17.6)	257 (9.0)		163 (10.9)	160 (10.7)	
II	1855 (45.2)	1139 (39.8)		621 (41.4)	621 (41.4)	
III	1446 (35.2)	1367 (47.8)		690 (46.0)	695 (46.4)	
IV	82 (2.0)	97 (3.4)		25 (1.7)	23 (1.5)	
Nodal status			<0.001			0.898
Node negative	3944 (96.0)	1861 (65.1)		1366 (91.1)	1364 (91.0)	
Node positive	164 (4.0)	999 (34.9)		133 (8.9)	135 (9.0)	
Size			<0.001			0.877
≤2 cm	2401 (58.4)	792 (27.7)		539 (36.0)	536 (35.8)	
2–4 cm	1333 (32.4)	1232 (43.1)		672 (44.8)	664 (44.3)	
>4 cm	374 (9.1)	836 (29.2)		288 (19.2)	299 (19.9)	
Surgical method			<0.001			0.162
Local tumor destruction	197 (4.8)	281 (9.8)		77 (5.1)	66 (4.4)	
Hysterectomy	3904 (95.0)	2551 (89.2)		1420 (94.7)	1426 (95.1)	
Pelvic exenteration	7 (0.2)	28 (1.0)		2 (0.1)	7 (0.5)	

CDS: cancer-directed surgery; RT: radiotherapy.

### Impact of local treatment modalities on survival

**Figure 1:**
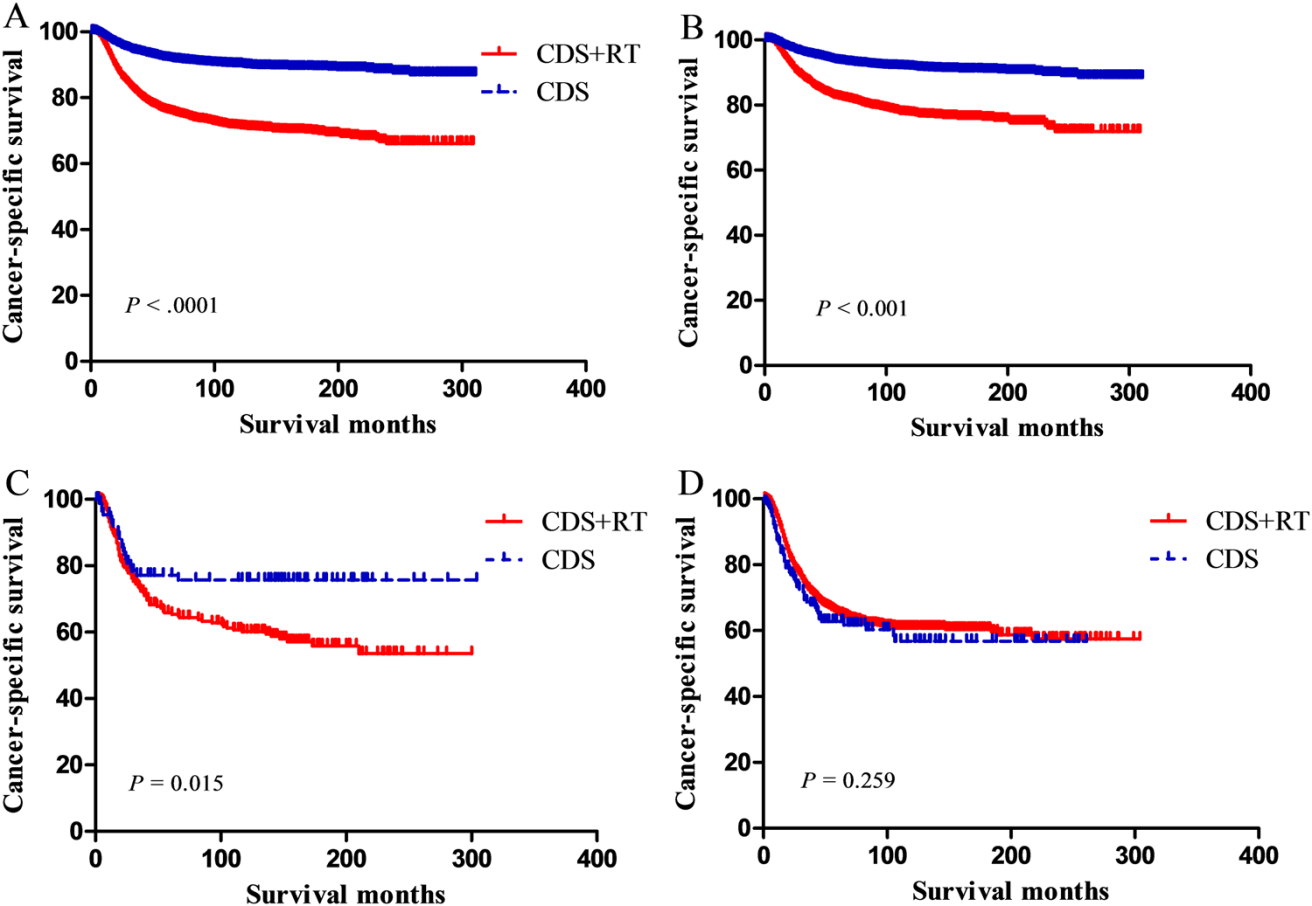
Cause-specific survival of patients with FIGO stage IB-IIB (**A**), IB (**B**), IIA (**C**) and IIB (**D**) with different local treatment modalities.

Kaplan-Meier analysis showed the impact of local treatment modalities on CSS for early stage cervical cancer patients (Fig. 1). Patients who received CDS alone had better CSS (*P* < 0.001; Fig. 1A) compared to patients who had CDS + RT. According to the NCCN guidelines, the preferred treatments for IB-IIB cervical cancer depend on disease stages. Thus, the prognostic effects of the different local treatment modalities according to FIGO stage were evaluated. For patients with IB and IIA cervical cancer, CDS was associated with better CSS (*P* < 0.001; Fig. 1B and C, respectively); nevertheless, for patients with IIB cervical cancer, no differences were observed in CSS according to different local treatment modalities (*P* = 0.259; Fig. 1D). According to this, we divided the data into two groups: one contains FIGO stage IB-IIA patients, the other FIGO stage IIB patients. Because there were both significant differences in the baseline characteristic of two groups, we performed two PSM analyses at a 1:1 ratio and a 5:1 ratio to erase significant difference of each variable, respectively (Table 3 and Table 4, respectively).

**Table 4:** Baseline characteristics of PSM cohorts for FIGO stage IIB.

Variable	Unmatched (complete) dataset			Matched (5:1) dataset		
	CDS + RT (%)	CDS (%)	P	CDS + RT (%)	CDS (%)	P
	(n = 1199)	(n = 190)		(n = 950)	(n = 190)	
Age			0.016			0.474
<50	661 (55.1)	87 (45.8)		462 (48.6)	87 (45.8)	
≥50	538 (44.9)	103 (54.2)		488 (51.4)	103 (54.2)	
Race			0.288			0.370
White	913 (76.1)	146 (76.8)		731 (76.9)	146 (76.8)	
Black	126 (10.5)	25 (13.2)		99 (10.4)	25 (13.2)	
Other	160 (13.3)	19 (10.0)		120 (12.6)	19 (10.0)	
Marital status			0.331			0.506
Single	295 (24.6)	53 (27.9)		243 (25.6)	53 (27.9)	
Married	904 (75.4)	137 (72.1)		707 (74.4)	137 (72.1)	
Histologic type			0.189			0.384
Squamous cell carcinoma	812 (67.7)	135 (71.1)		655 (68.9)	135 (71.1)	
Adenocarcinoma	139 (11.6)	12 (6.3)		97 (10.2)	12 (6.3)	
Adenosquamous carcinoma	88 (7.3)	15 (7.9)		76 (8.0)	15 (7.9)	
Others	160 (13.3)	28 (14.7)		122 (12.8)	28 (14.7)	
Primary site			0.232			0.810
Cervix uteri	899 (75.0)	152 (80.0)		765 (80.5)	152 (80.0)	
Endocervix	238 (19.8)	34 (17.9)		161 (16.9)	34 (17.9)	
Exocervix	25 (2.1)	1 (0.5)		12 (1.3)	1 (0.5)	
Overlapping lesion of cervix uteri	37 (3.1)	3 (1.6)		12 (1.3)	3 (1.6)	
Grade			0.110			0.743
I	73 (6.1)	6 (3.2)		33 (3.5)	6 (3.2)	
II	473 (39.4)	72 (37.9)		360 (37.9)	72 (37.9)	
III	602 (50.2)	98 (51.6)		506 (53.3)	98 (51.6)	
IV	51 (4.3)	14 (7.4)		51 (5.4)	14 (7.4)	
Nodal status			0.733			0.506
Node negative	672 (56.0)	109 (57.4)		520 (54.7)	109 (57.4)	
Node positive	527 (44.0)	81 (42.6)		430 (45.3)	81 (42.6)	
Size			0.379			0.552
≤2 cm	141 (10.8)	22 (11.2)		103 (10.8)	20 (10.5)	
2–4 cm	433 (33.1)	75 (38.1)		331 (34.8)	74 (38.9)	
>4 cm	733 (56.1)	100 (50.8)		51.6 (54.3)	96 (50.5)	
Surgical method			<0.001			0.081
Local tumor destruction	301 (25.1)	21 (11.1)		140 (14.7)	21 (11.1)	
Hysterectomy	852 (71.1)	165 (86.8)		764 (80.4)	165 (86.8)	
Pelvic exenteration	46 (3.8)	4 (2.1)		46 (4.8)	4 (2.1)	

CDS: cancer-directed surgery; RT: radiotherapy.

**Figure 2:**
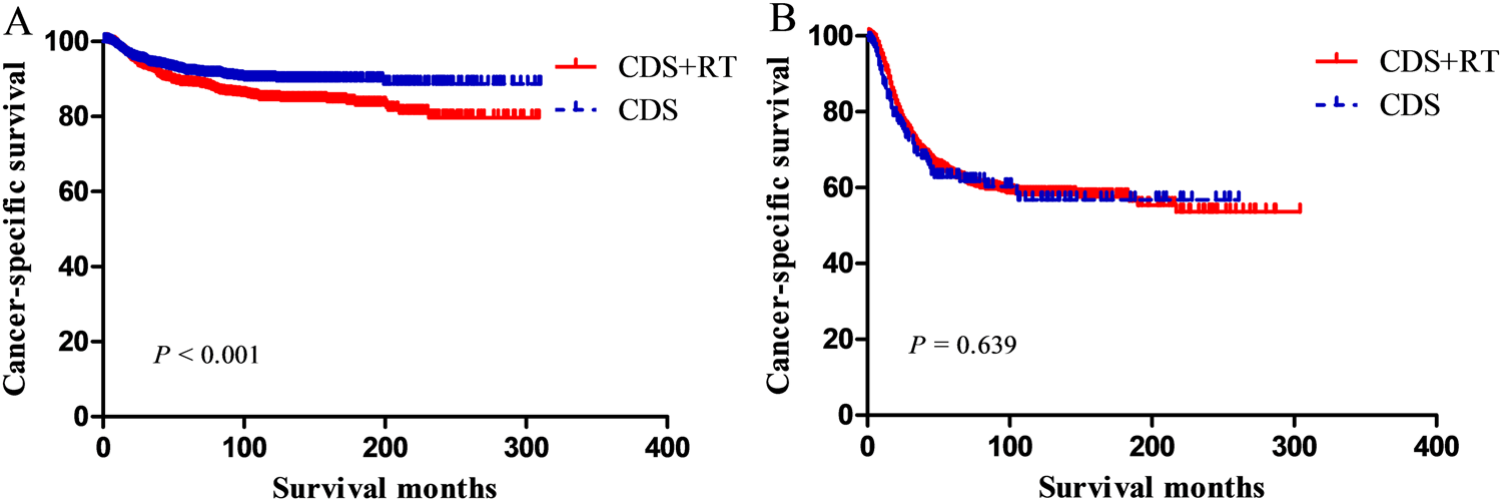
Cause-specific survival of patients with FIGO stage IB-IIA (**A**) and IIB (**B**) with different local treatment modalities in the matched data.

The 1:1 matching for CDS + RT versus CDS resulted in 1,499 matched pairs and a sample size of 2,998 patients (Table 3). The 5:1 matching for CDS + RT versus CDS resulted in 950 matched pairs and a sample size of 1,140 patients (Table 4). In the unmatched dataset, CDS was associated with better CSS in IB-IIA group (*P* < 0.001); nevertheless, no differences were observed in CSS according to different local treatment modalities in IIB group (*P* = 0.259). In the matched dataset, we obtained similar results: in IB-IIA group, CDS was still associated with better CSS (*P* < 0.001; Fig. 2A) and multivariate analysis demonstrated patients who received CDS + RT treatment had worse CSS (HR = 1.50; 95% CI = 1.18–1.90; *P* = 0.001); in IIB group, there was no difference between two treatment groups in CSS (*P* = 0.639; Fig. 2B).

## Discussion

In this study, we found that prognostic effects of the different local treatment modalities were greatly influenced by FIGO stage. On this basis, we further performed PSM analyses to adjust for biases from different baseline characteristics of two treatment groups. We demonstrated that CDS was associated with better CSS for patients IB-IIA cervical cancer. A retrospective study by Soisson *et al*. explained the reason why CDS outperformed CDS + RT. They reported that surgery alone had a better disease-free survival than combination therapy for patients with IB-IIA cervical cancer, but patients receiving adjuvant radiotherapy had several risk factors for recurrence, including lymphatic metastases, tumor involvement of the surgical margin, and large cervical lesions. Radiotherapy appears to reduce the incidence of pelvic recurrences but has no effect on distant failure of patients with high risks^7^.

Besides, no difference was observed comparing CDS and CDS + RT for patients with IIB cervical cancer in our study. Several retrospective studies suggested that radiotherapy combined with radical surgery could prolong the survival time for patients with IIB cervical cancer^8, 9^. However, the previous studies may have a few shortcomings. First, these studies are composed of limited cases. Second, because multivariate analysis in our study showed that race, histological subtypes, grade, nodal status and tumor size were all affected prognosis of cervical cancer patients, it is important to adjust for differences between baseline characteristics; nevertheless previous studies have significant differences between baseline characteristics. There was also prospective study that indicated similar results^11^. Unfortunately, the significant differences between baseline characteristics were still observed in prospective studies. By contrast, our study has overcome these shortcomings. First, this study has a large sample size which included a total of 8,357 patients who met our inclusion criteria. In the matched dataset, there were still 2,998 and 1,140 patients in FIGO stage IB-IIA and IIB group respectively. Second, our current study is the first to adjust for imbalance in the baseline characteristics between two treatment groups using PSM analysis. Thus, our study is statistically significant and innovative.

Zhou *et al*.^6^, using the SEER database, looked at the prognostic role of local treatment modalities in early stage small-cell carcinoma of the cervix (SCCC). They found that CDS was associated with better CSS in FIGO stage I SCCC; in FIGO stage II SCCC, no differences were observed in CSS. In our study, we adopted a more refined staging method to group patients into FIGO stage IB, IIA and IIB. We found that CDS was associated with better CSS in FIGO IB and IIA, and no differences were observed in FIGO IIB by subgroup analysis. Accordingly, we performed two PSM analyses regarding to IB-IIA and IIB respectively, which further confirmed our results. Furthermore, we investigate all subtypes of cervical cancer rather than simple subtype. Therefore, our study has larger application range and more reliability.

Although our study tried to overcome shortcomings of previous studies, it still had several potential limitations. First, SEER database lacks some important information regarding patient status, such as infection of human papilloma virus (HPV), comorbidities and economic conditions, that is apparently associated with prognosis^12, 13^. Second, due to the limitations of SEER database, the information of chemotherapy regimens and dose is unknown, that limits our ability to assess the clinical outcome of local treatment modalities. Third, although our study has measure the effects of different surgery methods divided by resection scope, beyond that, how many patients had lymphadenecthomy and the number of lymphonodes removed both have effects on surgical outcomes. Nevertheless, the above information was not collected into SEER database. Fourth, the same condition for radiotherapy. The technique used and radiotherapy doses cannot be assessed. Fifth, adjuvant radiotherapy is recommended when postoperative pathological examinations reveal risk factors for recurrence, including lymph node metastasis, parametrial invasion, deep stromal invasion, lymph vascular space involvement, and bulky tumor (tumor size >4 cm)^14^. Unfortunately, the information about these risks is incomplete.

In conclusion, combination therapy has been investigated in several settings for decades^15–17^, but our study demonstrated that compared with radical surgery alone, radical surgery combined with radiotherapy did not improve survival in early stage cervical cancer patients. Given the excessive financial burden and side effects from radiotherapy^7, 18, 19^, radical surgery alone is preferred treatment for early stage cervical cancer patients. Further prospective studies designed adequately with larger sample size are needed to confirm the results of this study, as well as to define optimal local management in early stage cervical cancer.

## Methods

### Patient selection in the SEER database

Patient data were obtained from the latest version of the SEER database as released in July 2016 (covering 18 registries, 1973–2013), by using SEER* Stat version 8.3.2. We have got the permission to access them on purpose of research only (Reference number: 12641-Nov2014). The data released by the SEER database do not require informed patient consent, and our study was approved by the Ethical Committee and Institutional Review Board of Soochow University. The methods were performed in accordance with the guidelines outlined in the Declaration of Helsinki.

We extracted cases of patients with a primary diagnosis of FIGO stage IB-IIB uterine cervical cancer (International Classification of Disease for Oncology, Third Edition) between 1988 and 2013. We excluded patients with more than one primary tumor, metastatic disease. Patients with unconfirmed or unknown tumor information were also excluded. The following covariates were collected from the database: age, race, marital status, histological subtypes, primary site, grade, FIGO stage, nodal status, size and surgical method. Relevant treatment-related data included cancer-directed surgery combined with radiotherapy (CDS + RT) and cancer-directed surgery (CDS) alone. In order to evaluate the overall effect of RT, we merge patients receiving either adjuvant or neoadjuvant RT into one group. Duration of follow-up, and cause of death described as due to cancer (CSS) were also included.

### Statistical analysis

The chi-squared test (or Fisher’s exact test, if appropriate) was used to analyze the differences between patients grouped by categorical variables. Clinical outcomes were compared between patients treated with CDS alone (referred to as CDS group) and CDS combined with RT (referred to as CDS + RT group). Survival curves were generated using Kaplan-Meier methods, and compared by log-rank test. Univariate and multivariate Cox regression analyses were used to analyze the independent risk factors for CSS. Only variables with statistical significance (*P* < 0.05) in univariate analysis were incorporated into multivariate analysis. Hazard ratios (HR) were calculated based on multivariable Cox proportional hazards models to estimate predictors of CSS. All CIs were stated at the 95% confidence level. Statistical significance was set at *P* < 0.05.

To adjust for differences between CDS + RT group and CDS group for FIGO stage IB-IIA and IIB patients, we performed two PSM analyses at a 1:1 ratio and a 5:1 ratio respectively. The PSM model was based upon age, race, marital status, histological subtypes, primary site, grade, nodal status, size and surgical method. The difference of each variable was considered significant if two-sided p-values less than 0.05. Statistical analyses were performed using SPSS statistical software package, version 22.0(SPSS Inc., Chicago, IL).
